# Association of Maternal Diabetes Mellitus and Polymorphisms of the *NKX2.5* Gene in Children with Congenital Heart Disease: A Single Centre-Based Case-Control Study

**DOI:** 10.1155/2020/3854630

**Published:** 2020-09-25

**Authors:** Mingyi Zhao, Jingyi Diao, Peng Huang, Jinqi Li, Yihuan Li, Yang Yang, Liu Luo, Senmao Zhang, Letao Chen, Tingting Wang, Ping Zhu, Jiabi Qin

**Affiliations:** ^1^Department of Pediatrics, The Third Xiangya Hospital, Central South University, Changsha, Hunan, China; ^2^Department of Epidemiology and Health Statistics, Xiangya School of Public Health, Central South University, Changsha, Hunan, China; ^3^Department of Cardiothoracic Surgery, Hunan Children's Hospital, Changsha, Hunan, China; ^4^Guangdong Cardiovascular Institute, Guangdong Provincial People's Hospital, Guangdong Academy of Medical Sciences, Guangzhou, Guangdong, China; ^5^National Health Commission Key Laboratory for Birth Defect Research and Prevention, Changsha, Hunan, China

## Abstract

**Background:**

Congenital heart disease (CHD) is one of the most common birth defects among newborns, accounting for a large proportion of infant mortality worldwide. However, the mechanisms remain largely undefinable. This study aimed to investigate the association of CHD in offspring of mothers with diabetes mellitus (DM) and single nucleotide polymorphisms (SNPs) of *NKX2.5*.

**Methods and Results:**

A case-control study of 620 mothers of CHD patients and 620 mothers of healthy children admitted to Hunan Children's Hospital from November 2017 to December 2019 was conducted. We collected the mothers' information by questionnaire and detected children's *NKX2.5* variants with a MassARRAY system. The interaction coefficient (*γ*) was used to quantify the estimated gene-environment interactions. Univariate and multivariate analyses both showed that the infants had a higher risk of CHD if their mothers had a history of DM, including gestational DM (GDM) during this pregnancy (adjusted odds ratio [aOR = 4.98]), GDM in previous pregnancies (aOR = 4.30), and pregestational DM (PGDM) in the 3 months before this pregnancy (aOR = 6.78). Polymorphisms of the *NKX2.5* gene at rs11802669 (C/C vs. T/T: aOR = 4.97; C/T vs. T/T: aOR = 2.15) and rs2277923 (T/T vs. C/C, aOR = 1.74; T/C vs. C/C, aOR = 1.61) were significantly associated with the risk of CHD in offspring. In addition, significant interactions between maternal DM and *NKX2.5* genetic variants at rs11802669 (aOR = 8.12) and rs2277923 (aOR = 17.72) affecting the development of CHD were found.

**Conclusions:**

These results suggest that maternal DM, *NKX2.5* genetic variants, and their interactions are significantly associated with the risk of CHD in offspring.

## 1. Introduction

Congenital heart disease (CHD) is one of the most common birth defects among newborns, accounting for a large proportion of infant mortality worldwide. As a rough estimate, approximately 8 infants in every 1000 are born with CHD [1]. The pathogenesis of CHD, however, is very complicated and different from other congenital diseases, and its exact causes are largely undefinable [2].

Approximately 2% of CHD can be attributed to known environmental factors, of which maternal diabetes mellitus (DM) is a well-accepted risk factor [3]. Gestational diabetes mellitus (GDM) poses significant risks of immediate and long-term health for the mother and foetus, affecting 3%-25% of pregnancies worldwide [4]. Studies have demonstrated that glycaemic control during pregnancy has a clear link to foetal malformations, and maternal DM appears to induce offspring malformation before the seventh week of gestation [5]. One hypothesis is that the abnormal glucose levels in maternal DM disrupt the expression of regulatory genes in the embryo, resulting in embryotoxic apoptotic cellular changes [6].

Over 40 genes have been implicated in the development of CHD [7]. Examples of CHD-causing mutations are those in *NKX2.5*, a transcriptional regulator during early embryonic heart development [8, 9]. Experiments have shown that *NKX2.5* is essential for the formation and maturation of the heart as well as the conduction system. Its absence in mice results in embryonic lethality and arrested heart development [10]. In addition, more than 40 mutations of *NKX2.5* have been found in CHD cases, which may reduce the transcriptional activity of *NKX2.5* and affect cardiac development [11]. Several animal experiments have shown that *NKX2.5* may be involved in the pathogenesis of embryonic congenital heart disease caused by gestational diabetes and that in a rat model, the expression of *NKX2.5* decreased during heart development in foetuses of mothers with gestational diabetes [12–14].

Accordingly, we conducted a case-control study including 620 mothers of CHD patients and 620 mothers of healthy children, collecting mothers' information by questionnaire and detecting children's *NKX2.5* variants with a MassARRAY system, to study the association of maternal DM, *NKX2.5* variants and their interaction with CHD in offspring to provide a new direction for the prevention of CHD.

## 2. Methods

### 2.1. Recruitment of Study Participants

We conducted a case-control study to investigate the role of maternal DM and *NKX2.5* SNPs in the aetiology of CHD in offspring. Recruitment was conducted by the Hunan Children's Hospital from November 2017 to December 2019. Children with CHD and their parents who were seen in this hospital were identified as the case group. All CHD patients were diagnosed using ultrasonography, and their diagnosis was confirmed by surgery. Children without any congenital malformation after a medical examination and their parents were identified as the control group. The study participants were recruited at 2 clinics from this hospital. The case group was recruited from the Department of Cardiothoracic Surgery, which provides diagnosis, treatment, surgery, and management for CHD, and the control group was recruited from the Department of Child Healthcare after health counselling or a medical examination. The controls were selected from the same hospital during the same study period as the cases. This study was approved by the ethics committee of the Xiangya School of Public Health of Central South University, and written informed consent was obtained from all mothers. This study was registered in the Chinese Clinical Trial Registry Center (registration number: ChiCTR1800016635).

### 2.2. Inclusion Criteria

In this study, the exposures of interest were maternal DM and children's genetic variants of the *NKX2.5* gene. DM was identified based on the following International Classification of Diseases and Related Health Problems, Tenth Revision (ICD-10) code: O24.4. Three types of maternal DM were included: gestational DM (GDM) during this pregnancy, GDM in previous pregnancies, and pregestational DM (PGDM) in the 3 months before this pregnancy. The outcomes of interest were CHD in the offspring, including seven subtypes: atrial septal defect (ASD), ventricular septal defect (VSD), atrioventricular septal defect (AVSD), patent ductus arteriosus (PDA), aortopulmonary septal defect (APSD), tetralogy of Fallot (TOF), and complete transposition of the great arteries (CTOGA). All CHD patients were diagnosed using ultrasonography, and their diagnosis was confirmed by surgery.

To reduce recall bias, mothers with children under 1 year old were recruited and asked to complete the same questionnaire in the same way by professionally trained investigators who were not informed in advance of the classification of the cases and controls. Additionally, this study was aimed at mothers of Han Chinese descent with singleton pregnancies. Eligible mothers needed to be able to complete the questionnaire and provide informed consent and blood samples. Nonsyndromic CHDs were of interest, and patients with other organ malformations or known abnormalities were excluded. Children without any congenital malformation after a medical examination served as the controls.

### 2.3. Information Collection

A standardized questionnaire was used to assess the history of maternal DM, including GDM during this pregnancy, GDM in previous pregnancies, and PGDM in the 3 months before this pregnancy. In addition, we consulted the Maternal and Child Health Manual and maternal medical records to further confirm the corresponding information on maternal DM history. In China, each pregnant woman is provided with a Maternal and Child Health Manual in which to record their basic demographic characteristics, behavioural habits, illnesses, and the results of various medical examinations during pregnancy. Another major exposure of interest in this study was the SNPs of the *NKX2.5* gene at rs6882776, rs118026695, rs2277923, and rs703752, which are described below.

When evaluating the association of maternal DM and *NKX2.5* SNPs with the risk of CHD in offspring, some other confounding factors needed to be considered. Therefore, we collected the following information about the mothers: (1) demographic characteristics, (2) abnormal pregnancy history, (3) family history, (4) personal medical history, (5) lifestyle and habits, (6) history of exposure to environmental hazardous substances, and (7) medical history during this pregnancy.

### 2.4. *NKX2.5* SNP Sequencing

The genetic loci rs6882776, rs118026695, rs2277923, and rs703752 of the *NKX2.5* gene, which have been widely studied previously, were selected as candidate loci for this study [15–17]. Three to five millilitres of peripheral venous blood from the children was used for genotyping. Blood samples were collected in EDTA-treated (ethylenediamine tetraacetic acid) anticoagulant tubes and then immediately centrifuged into plasma and white blood cells. White blood cells were separated and stored at -80°C until genotyping was performed. DNA was extracted from blood cells by the QIAamp DNA Mini Kit (Qiagen, Valencia, CA, USA) according to the manufacturer's standard protocol and dissolved in sterile TBE (Tris-borate-EDTA) buffer. The concentration and purity of the DNA solution were detected by ultraviolet spectrophotometry to ensure that the DNA was eligible as a template for polymerase chain reaction (PCR).

Primer designs, amplification conditions, and expected product sizes for *NKX2.5* have been described by previous studies [15–17]. The polymorphisms of the *NKX2.5* gene at rs6882776, rs118026695, rs2277923, and rs703752 were tested using a matrix-assisted laser desorption and ionization time-of-flight mass spectrometry MassARRAY system (Agena iPLEXassay, San Diego, CA, USA). The error rate for genotyping was less than 5%. The experimenters who performed the genotyping were not informed in advance of the status of the control or case groups. Each sample was retyped and double-checked to ensure the reliability of the experiments.

For rs6882776, three genotypes, including the homozygous wild-type G/G, the heterozygous variant T/A, and the homozygous variant A/A, were identified. For rs118026695, three genotypes were identified: the homozygous wild-type T/T, the heterozygous variant C/T, and the homozygous variant C/C. For rs2277923, three genotypes were identified: the homozygous wild-type T/T, the heterozygous variant T/C, and the homozygous variant C/C. For rs703752, three genotypes were identified: the homozygous wild-type C/C, the heterozygous variant C/A, and the homozygous variant A/A.

### 2.5. Statistical Analysis

Means and standard deviations were reported for normally distributed continuous variables. For continuous variables that did not seem normally distributed and categorical variables, quartiles (%) were reported. In the univariate analysis, the Pearson chi-squared test or Fisher's exact test was used to compare categorical variable data; the Wilcoxon rank sum test was used for nonnormally distributed continuous variables.

*NKX2.5* SNPs were analysed for deviations from Hardy-Weinberg equilibrium (HWE). The correlation analysis for CHD and maternal DM or *NKX2.5* SNPs was performed by univariate and multivariate logistic regression analysis, and odds ratios (ORs) and their 95% confidence intervals (CIs) were used to assess the level of association. The ORs were adjusted (aORs) using the factors with *P* values less than 0.05 in Table 1. We used logistic regression and controlled for potential confounding factors to examine the main effects and interactive effects of the gene-environment interaction of the *NKX2.5* gene and maternal DM on the risk of CHD in offspring.

In the logistic regression model, the confounding factors, maternal DM, and *NKX2.5* SNPs were set as independent variables (covariates), and the effects were expressed as ORs with 95% CIs. Offspring diagnostic results were set as dependent variables (binary outcomes). Models of the gene-environment interactions and their implications referred to the method described by Wallace [17]. The interaction coefficient (*γ*) was calculated as a function of the regression coefficient (*β*) from the logistic regression analysis (e.g., *γ*1 = *β*e∗g/*β*e and *γ*2 = *β*e∗g/*β*g for gene-environment interaction) and was used to evaluate the interaction [18]. When all *γ* values were greater than 1, there was a positive interaction; when all *γ* values were less than 1, there was a negative interaction; and when the *γ* values were equal to 1, there was no interaction.

In this study, we examined the risk of total CHD instead of the risk of specific subtypes due to the limited sample size. All data were analysed using SPSS (IBM SPSS Statistics for Macintosh, Version 26.0, Armonk, NY: IBM Corp). *P* < 0.05 was considered statistically significant.

## 3. Results

### 3.1. Comparison of Baseline Characteristics

We analysed seven aspects of the 1240 questionnaires (620 control groups and 620 case groups) (Table 1). The pregnancy age (*P* = 0.003), educational level (*P* ≤ 0.001), and family annual income (*P* ≤ 0.001) of women in the case group were significantly lower than those in the control group. However, there was no significant difference in BMI (*P* = 0.104). Moreover, the incidences of history of abnormal pregnancy (except for spontaneous abortion, embryo damage, and placenta previa), familial congenital malformations, personal medical history (except for history of congenital malformation), history of exposure to environmental hazardous substances (except for house renovation), and medication history during this pregnancy (except for folic acid) were significantly higher in the case group than in the control group. There were also significant differences in lifestyle between the two groups. The factors with *P* values less than 0.05 were included in the follow-up multivariate analysis.

### 3.2. Maternal DM Increases the Risk of Offspring CHD

During this pregnancy, 10.2% of women had PGDM, and 11.0% had GDM in the case group, both of which were significantly higher than the respective percentages of women in the control group (*P* < 0.001) (Table 2). There were also more women with GDM during previous pregnancies in the case group (*P* ≤ 0.001). The unadjusted ORs for these three types of maternal DM were 2.61, 3.03, and 4.01, respectively, which demonstrated that if the mother had a history of DM, there was a higher risk for their offspring to develop CHD. Next, the relationship between maternal DM and offspring CHD was analysed by multivariate analysis (Table 3). Maternal DM was classified into 3 types, including GDM during this pregnancy, GDM in previous pregnancies, and PGDM in the 3 months before this pregnancy, and the risk factors in Table 1, such as age, educational level, and medical history, were adjusted in the analysis of these three types. There was a significant correlation between maternal DM and offspring CHD (*P* ≤ 0.001), and maternal DM increased the risk of offspring CHD (aOR = 4.98, 4.30, 6.78, respectively).

### 3.3. *NKX2.5* Variation Increases the Risk of CHD in Offspring

Univariate analysis showed that there were associations between CHD and the genotype or allele frequencies of any polymorphism of rs118026695, rs2277923, and rs703752 in *NKX2.5* (Table 4). Next, we performed multivariate analysis (Table 5). The homozygous genotypes with a larger number of control groups were used as the control genotype. The confounding factors in Table 1 were adjusted in the analysis. Mutations of rs118026695 and rs2277923 were demonstrated to be associated with CHD.

T/T, C/T, and C/C genotypes were detected at rs118026695, and their frequency distributions were significantly different between the control and case groups (*P* ≤ 0.001). The logistic regression analysis revealed that C/T (*P* ≤ 0.001, aOR = 2.15, 95% CI) and C/C (*P* = 0.023, aOR = 4.97, 95% CI) were positively correlated with CHD. The frequency distribution of 2 alleles (C, T) also showed a significant difference between the control and case groups (*P* ≤ 0.001), and the logistic regression analysis showed that the C allele was positively correlated with CHD (*P* ≤ 0.001, OR = 1.81, 95% CI).

C/C, T/C, and T/T genotypes were detected at rs2277923, and their frequency distributions were significantly different between the control and case groups (*P* ≤ 0.001). The logistic regression analysis revealed that T/C (*P* = 0.004, aOR = 1.61, 95% CI) and T/T (*P* = 0.021, aOR = 1.74, 95% CI) were positively correlated with CHD. The frequency distribution of 2 alleles (C, T) also showed a significant difference between the control and case groups (*P* ≤ 0.001), and the logistic regression analysis showed that the T allele was positively correlated with CHD (*P* ≤ 0.001, OR = 1.38, 95% CI).

### 3.4. Analysis of the Interaction of Maternal DM and *NKX2.5* in the Development of CHD in Offspring

Based on the above results, three types of maternal DM and the *NKX2.5* SNPs at rs11802669 and rs2277923 were chosen to undergo further analysis (Table 6). We analysed their interaction and found that the interactions between maternal GDM during this pregnancy and C/T at rs11802669 (*P* = 0.008, aOR = 8.12, 95% CI) or T/C at rs2277923 (*P* ≤ 0.001, aOR = 17.72, 95% CI) increased the risk of CHD in offspring.

## 4. Discussion

Cardiac development is a complicated process involving the dual roles of genes and the environment. A classic example of a perturbed maternal environment that is closely associated with CHD is maternal GDM [19]. GDM, with its hyperglycaemic milieu during the first trimester, is related to diabetic embryopathy, affecting the heart, great vessels, and neural tube [20]. GDM during the latter half of pregnancy is related to foetal macrosomia, cardiomyopathy, and an increased incidence of perinatal complications and mortality [21]. Maternal DM was found to be a risk factor for offspring CHD in this study, which suggests the role of glucose in the causal pathway. Studies have indicated that the offspring of women with DM have similar risks for most types of CHD, and the increased risk of CHDs for them exceeded the increased risk of noncardiac diseases associated with maternal DM, which might be due to the impacts of maternal DM on general cardiac development very early in embryogenesis [22, 23]. This is consistent with our results and implies that some degree of glycaemic control before and during pregnancy is necessary. In addition, obesity has been associated with a small increase in the risk of CHD [24]. Our study, however, did not support this conclusion. There was no significant difference in BMI between the control and case groups, which indicated that there were other vital factors impacting offspring CHD, especially for the women who developed DM during this pregnancy.

Many studies show that the occurrence and development of CHD might be associated with changes in transcription factors in offspring. Over the years, family-based studies have identified mutations in transcription factor genes controlling heart development, including NKX2.5, GATA4, and TBX5 [25]. These mutations are often observed in patients with CHD but are rarely found in patients without CHD [26]. In our study, *NKX2.5* was proven to be associated with the incidence of CHD, and mutations at rs118026695 and rs2277923 were demonstrated to increase the risk of CHD in the univariate and multivariate analyses.

*NKX2.5* is a member of the NK-2 family of homeodomain-containing transcription factors, which are highly conserved in many organisms [27]. It is a transcriptional regulator during early embryonic heart development and essential for the formation and maturation of the heart as well as the conduction system. Experiments indicate that the absence of *NKX2.5* in mice results in embryonic lethality and arrested heart development [10]. In addition, more than 40 mutations of *NKX2.5* have been found in CHD cases, and the 63A>G (rs2277923) polymorphism was one of the most intensively investigated sites [28]. Several studies have found that this polymorphism is significantly associated with CHD risk [11] and that this mutation is capable of changing mRNA structure and stability instead of the amino acid sequence of the encoded protein [29]. Moreover, the transcriptional activity of *NKX2.5* is also related to CHD. Ouyang et al. revealed that the 63A>G mutation significantly reduced the transcriptional activity of *NKX2.5* by 20%, which might account for its association with CHD [11]. Some opposite results, however, were also found in CHD studies in which the SNPs of *NKX2.5* at rs2277923 were not associated with the incidence of CHD in China [30]. The difference in results might be due to the choice of region, race, and CHD subtypes. Thus, further studies with large sample sizes are required to clarify this association.

The incidence of CHD is a result of both genetic and environmental factors. Their interactions are profoundly heterogeneous but may operate on common pathways. Nina et al. thought that glucose itself might exert a teratogenic effect via a signalling pathway regulating insulin sensitivity, which is also a key mediator of embryogenesis and early development [22]. Experimental studies found that hyperglycaemia in early pregnancy affected regulatory gene expression in the embryo for genes, such as Bmp4, Msx1, and Pax3, leading to cardiac neural crest cell death and an increased risk of CHD [31]. Whether the expression of *NKX2.5*, an important transcriptional regulator in early cardiac development, is affected by hyperglycaemia is worthy of further experimental investigation. In addition, *NKX2.5* is expressed in the first heart field (FHF) and the second heart field (SHF), the two distinct sources of cardiac progenitor cells contributing to different parts of the heart [32]. In mice, *NKX2.5* repression of Bmp2/Smad1 signalling regulated SHF proliferation and outflow tract morphology [33]. In the cardiac fields of *NKX2.5* mutants, failed SHF proliferation and OFT truncation were also found [34]. Similarly, hyperglycaemia was found to take part in the development of SHF, and the association with maternal diabetes mellitus was much stronger for anterior second heart field defects than for posterior second heart field defects when grouping the outflow tract malformations and the inflow tract malformations [19]. Based on the above, the interaction of maternal DM and *NKX2.5* genetic variants deserves further exploration. To the best of our knowledge, this is the first study to explore the association of the interaction of maternal DM and *NKX2.5* genetic variants with the risk of CHD in offspring.

Finally, we acknowledge important limitations in our study. This is a retrospective study, and recall bias is inevitable, even though we took measures to reduce it in sample selection and information collection. In addition, this study was aimed at Han Chinese individuals and was restricted geographically, so different ethnic populations and large sample sizes are required to confirm our results. Finally, the outcome of interest in this study was total CHD instead of specific CHD subtypes, which might contribute to the inaccuracy of the results.

## 5. Conclusion

In this study, we investigated the risk factors associated with CHD. We demonstrated that maternal DM, *NKX2.5* variants, and their interaction were significantly associated with CHD in offspring. With the aim of preventing CHD, there is a need for women to control their glycaemic index during pregnancy and to screen for *NKX2.5* mutations in their children if necessary.

## Figures and Tables

**Table 1:** Baseline characteristics comparison between control group and case group.

Variables	Control group (*n* = 620)	Case group (*n* = 620)	Univariable analysis
Demographic characteristics			
Maternal age at this pregnancy (years)	28.5 ± 4.7	28.0 ± 5.4	
≤24	124 (20.0%)	176 (28.4%)	*χ* ^2^ = 13.755; *P* = 0.003
25-29	261 (42.1%)	235 (37.9%)	
30-34	152 (24.5%)	122 (19.7%)	
≥35	83 (13.4%)	87 (14.0%)	
Education level			
Less than primary or primary	7 (1.1%)	87 (14.0%)	*Z* = 14.342; *P* ≤ 0.001
Junior high school	117 (18.9%)	263 (42.4%)	
Senior middle school	217 (35.0%)	167 (26.9%)	
College or above	279 (45.0%)	103 (16.6%)	
Body mass index before this pregnancy			
<18.5	156 (25.8%)	112 (18.3%)	*Z* = 1.625; *P* = 0.104
18.5-23.99	340 (56.3%)	404 (65.9%)	
≥24	108 (17.9%)	97 (15.8%)	
Family's annual income in the past 1 year (RMB)			
≤50,000	179 (28.9%)	494 (79.7%)	*Z* = 17.785; *P* ≤ 0.001
60,000-100,000	267 (43.1%)	92 (14.8%)	
110,000-150,000	57 (9.2%)	12 (1.9%)	
≥160,000	117 (18.9%)	22 (3.5%)	
Residence location			
Rural areas	342 (55.2%)	444 (71.6%)	*χ* ^2^ = 36.153; *P* ≤ 0.001
Urban areas	278 (44.8%)	176 (28.4%)	
Abnormal pregnancy history			
Spontaneous abortion			
No	560 (90.3%)	545 (87.9%)	*χ* ^2^ = 1.870; *P* = 0.171
Yes	60 (9.7%)	75 (12.1%)	
Induced abortion or labor			
No	428 (69.0%)	363 (58.5%)	*χ* ^2^ = 14.751; *P* ≤ 0.001
Yes	192 (31.0%)	257 (41.5%)	
Fetal death or stillbirth			
No	618 (99.7%)	584 (94.2%)	*χ* ^2^ = 31.383; *P* ≤ 0.001
Yes	2 (0.3%)	36 (5.8%)	
Premature delivery			
No	614 (99.0%)	603 (97.3%)	*χ* ^2^ = 5.360; *P* = 0.021
Yes	6 (1.0%)	17 (2.7%)	
Low birth weight			
No	617 (99.5%)	603 (97.3%)	*χ* ^2^ = 9.961; *P* = 0.002
Yes	3 (0.5%)	17 (2.7%)	
Neonatal death			
No	620 (100.0%)	603 (97.3%)	*χ* ^2^ = 17.236; *P* ≤ 0.001
Yes	0 (0.0%)	17 (2.7%)	
Embryo damage			
No	610 (98.4%)	604 (97.4%)	*χ* ^2^ = 1.414; *P* = 0.234
Yes	10 (1.6%)	16 (2.6%)	
Hypertension of pregnancy			
No	611 (98.5%)	577 (93.1%)	*χ* ^2^ = 23.204; *P* ≤ 0.001
Yes	9 (1.5%)	43 (6.9%)	
Placenta previa			
No	615 (99.2%)	613 (98.9%)	*χ* ^2^ = 0.337; *P* = 0.562
Yes	5 (0.8%)	7 (1.1%)	
Placental abruption			
No	620 (100.0%)	620 (100.0%)	
Yes	0 (0.0%)	0 (0.0%)	
Premature rupture of membranes			
No	620 (100.0%)	615 (99.2%)	*P* = 0.062 (Fisher's exact test)
Yes	0 (0.0%)	0.8%	
Anemia during pregnancy			
No	598 (96.5%)	554 (89.4%)	*χ* ^2^ = 23.681; *P* ≤ 0.001
Yes	22 (3.5%)	66 (10.6%)	
Family history			
Consanguineous marriages			
No	617 (99.5%)	599 (96.6%)	*χ* ^2^ = 13.766; *P* ≤ 0.001
Yes	3 (0.5%)	21 (3.4%)	
Congenital malformations			
No	615 (99.2%)	584 (94.2%)	*χ* ^2^ = 24.241; *P* ≤ 0.001
Yes	5 (0.8%)	36 (5.8%)	
Personal medical history			
Personal history of congenital malformations			
No	617 (99.5%)	611 (98.5%)	*χ* ^2^ = 3.029; *P* = 0.082
Yes	3 (0.5%)	9 (1.5%)	
Cold history in the 3 months before this pregnancy			
No	548 (88.4%)	499 (80.5%)	*χ* ^2^ = 14.734; *P* ≤ 0.001
Yes	72 (11.6%)	121 (19.5%)	
Fever history in the 3 months before this pregnancy			
No	608 (98.1%)	569 (91.8%)	*χ* ^2^ = 25.435; *P* ≤ 0.001
Yes	12 (1.9%)	51 (8.2%)	
Cold history during this pregnancy			
No	488 (78.7%)	413 (66.6%)	*χ* ^2^ = 22.836; *P* ≤ 0.001
Yes	132 (21.3%)	207 (33.4%)	
Fever history during this pregnancy			
No	601 (96.9%)	561 (90.5%)	*χ* ^2^ = 21.890; *P* ≤ 0.001
Yes	19 (3.1%)	59 (9.5%)	
Lifestyle and habit			
History of active smoking in the 3 months before this pregnancy			
No	607 (97.9%)	584 (94.2%)	*χ* ^2^ = 11.240; *P* = 0.001
Yes	13 (2.1%)	36 (5.8%)	
History of passive smoking in the 3 months before this pregnancy			
No	393 (63.4%)	293 (47.3%)	*χ* ^2^ = 32.628; *P* ≤ 0.001
Yes	227 (36.6%)	327 (52.7%)	
Drinking history in the 3 months before this pregnancy			
No	577 (93.1%)	539 (86.9%)	*χ* ^2^ = 12.939; *P* ≤ 0.001
Yes	43 (6.9%)	81 (13.1%)	
History of drinking tea in the 3 months before this pregnancy			
No	503 (81.1%)	542 (87.4%)	*χ* ^2^ = 9.256; *P* = 0.002
Yes	117 (18.9%)	78 (12.6%)	
History of drinking coffee in the 3 months before this pregnancy			
No	592 (95.5%)	565 (91.1%)	*χ* ^2^ = 9.413; *P* = 0.002
Yes	28 (4.5%)	55 (8.9%)	
History of chewing betel nut in the 3 months before this pregnancy			
No	608 (98.4%)	593 (95.6%)	*χ* ^2^ = 7.995; *P* = 0.005
Yes	10 (1.6%)	27 (4.4%)	
Frequency of cosmetics use in the 3 months before this pregnancy			
Never	360 (58.1%)	293 (47.3%)	*Z* = 4.993; *P* ≤ 0.001
Sometime	174 (28.1%)	150 (24.2%)	
Often	40 (6.5%)	105 (16.9%)	
Every day	46 (7.4%)	72 (11.6%)	
Did you keep pets in the 3 months before this pregnancy?			
No	573 (92.4%)	537 (86.6%)	*χ* ^2^ = 11.137; *P* = 0.001
Yes	47 (7.6%)	83 (13.4%)	
Did you often dye or perm your hair in the 3 months before this pregnancy?			
No	584 (94.2%)	547 (88.2%)	*χ* ^2^ = 13.770; *P* ≤ 0.001
Yes	36 (5.8%)	73 (11.8%)	
Exposure history to environmental hazardous substance			
Was there a factory near place of residence that discharges environmentally harmful substances?			
No	574 (92.6%)	503 (81.1%)	*χ* ^2^ = 35.607; *P* ≤ 0.001
Yes	46 (7.4%)	117 (18.9%)	
Was there a traffic road or a noisy factory near where you live (noise exposure)?			
No	506 (81.6%)	446 (71.9%)	*χ* ^2^ = 16.282; *P* ≤ 0.001
Yes	114 (18.4%)	174 (28.1%)	
Was your house newly renovated in the 3 months before this pregnancy?			
No	567 (91.5%)	572 (92.3%)	*χ* ^2^ = 0.269; *P* = 0.604
Yes	53 (8.5%)	48 (7.7%)	
Medicine history in this pregnancy			
Folate			
Yes	577 (93.1%)	526 (84.8%)	*χ* ^2^ = 21.344; *P* ≤ 0.001
No	43 (6.9%)	94 (15.2%)	
Macrolide antibiotics			
No	603 (97.3%)	569 (91.8%)	*χ* ^2^ = 17.986; *P* ≤ 0.001
Yes	17 (2.7%)	51 (8.2%)	
Antidepressants			
No	586 (94.5%)	551 (88.9%)	*χ* ^2^ = 12.971; *P* ≤ 0.001
Yes	34 (5.5%)	69 (11.1%)	
Fetal-prevention drugs			
No	539 (86.9%)	487 (78.5%)	*χ* ^2^ = 15.271; *P* ≤ 0.001
Yes	81 (13.1%)	133 (21.5%)	

**Table 2:** Univariate analysis of maternal DM and offspring CHD.

Maternal DM	Control group (*n* = 620)	Case group (*n* = 620)	Univariable analysis	Unadjusted OR (95% CI)
GDM during this pregnancy				
No	592 (95.5%)	552 (89.0%)	*χ* ^2^ = 18.065; *P* ≤ 0.001	1
Yes	28 (4.5%)	68 (11.0%)		2.61 (1.65-4.11)∗
GDM during previous pregnancy experience				
No	595 (96.0%)	550 (88.7%)	*χ* ^2^ = 23.084; *P* ≤ 0.001	1
Yes	25 (4.0%)	70 (11.3%)		3.03 (1.89-4.85)^∗^
PGDM in the 3 months before this pregnancy				
No	603 (97.3%)	557 (89.8%)	*χ* ^2^ = 28.274; *P* ≤ 0.001	1
Yes	17 (2.7%)	63 (10.2%)		4.01 (2.32-6.94)^∗^

DM: diabetes mellitus; CHD: congenital heart disease; GDM: gestational diabetes mellitus; PGDM: pregestational diabetes mellitus; OR: odds ratio; CI: confidence interval. ^∗^Statistically significant (*a* = 0.05).

**Table 3:** Multivariate analysis of maternal DM and offspring CHD.

Maternal DM	B	S.E	Wals	*P* value	Adjusted OR (95% CI)^∗^
GDM during this pregnancy (yes vs. no)	1.609	0.340	22.326	0.001	4.98 (2.56-9.74)
GDM in previous pregnancy experiences (yes vs. no)	1.458	0.334	19.117	0.001	4.30 (2.24-8.27)
PGDM in the 3 months before this pregnancy (yes vs. no)	1.914	0.385	24.676	0.001	6.78 (3.19-14.43)

DM: diabetes mellitus; CHD: congenital heart disease; GDM: gestational diabetes mellitus; PGDM: pregestational diabetes mellitus; OR: odds ratio; CI: confidence interval. ^∗^Adjusted for factors with statistical differences in Table 1.

**Table 4:** Univariate analysis of *NKX2.5* gene polymorphism and CHD.

SNP of *NKX2.5* gene	Control group (*n* = 620)	Case group (*n* = 620)	Univariate analysis	Unadjusted OR (95% CI)
Genotype at rs6882776				**0.98 (0.83-1.16)**
A/A	216 (34.8%)	209 (33.7%)	*χ* ^2^ = 1.688; *P* = 0.430	1
G/A	308 (49.7%)	328 (52.9%)		1.10 (0.86-1.41)
G/G	96 (15.5%)	83 (13.4%)		0.89 (0.63-1.27)
Recessive model at rs6882776				
A/A	216 (34.8%)	209 (33.7%)	*χ* ^2^ = 0.175; *P* = 0.675	1
G/A+G/G	404 (65.2%)	411 (66.3%)		1.05 (0.83-1.33)
Dominant model at rs6882776				
A/A+ G/A	524 (84.5%)	537 (86.6%)	*χ* ^2^ = 1.103; *P* = 0.294	1
G/G	96 (15.5%)	83 (13.4%)		0.84 (0.61-1.16)
Allele at rs6882776				
A	740 (59.7%)	746 (60.2%)	*χ* ^2^ = 0.060; *P* = 0.806	1
G	500 (40.3%)	494 (39.8%)		0.98 (0.84-1.15)
Genotype at rs118026695				**1.83 (1.42-2.36)** ^∗^
T/T	510 (82.3%)	443 (71.5%)	*χ* ^2^ = 22.949; *P* ≤ 0.001	1
C/T	107 (17.3%)	164 (26.5%)		1.77 (1.34-2.32)^∗^
C/C	3 (0.5%)	13 (2.1%)		4.99 (1.41-17.62)^∗^
Recessive model at rs118026695				
T/T	510 (82.3%)	443 (71.5%)	*χ* ^2^ = 20.352; *P* ≤ 0.001	1
C/T+C/C	110 (17.7%)	177 (28.5%)		1.85 (1.41-2.43)^∗^
Dominant model at rs118026695				
T/T+C/T	617 (99.5%)	607 (97.9%)	*χ* ^2^ = 6.332; *P* = 0.012	1
C/C	3 (0.5%)	13 (2.1%)		4.41 (1.25-15.53)^∗^
Allele at rs118026695				
T	1127 (90.9%)	1050 (84.7%)	*χ* ^2^ = 22.291; *P* ≤ 0.001	1
C	113 (9.1%)	190 (15.3%)		1.81 (1.41-2.31)^∗^
Genotype at rs2277923				**1.38 (1.17-1.62)** ^∗^
C/C	291 (46.9%)	217 (35.0%)	*χ* ^2^ = 18.268; *P* ≤ 0.001	1
T/C	254 (41.0%)	310 (50.0%)		1.64 (1.29-2.08)^∗^
T/T	75 (12.1%)	93 (15.0%)		1.66 (1.17-2.36)^∗^
Recessive model at rs2277923				
C/C	291 (46.9%)	217 (35.0%)	*χ* ^2^ = 18.260; *P* ≤ 0.001	1
T/C+T/T	329 (53.1%)	403 (65.0%)		1.64 (1.31-2.06)^∗^
Dominant model at rs2277923				
C/C+T/C	545 (87.9%)	527 (85.0%)	*χ* ^2^ = 2.231; *P* = 0.135	1
T/T	75 (12.1%)	93 (15.0%)		1.28 (0.93-1.78)
Allele at rs2277923				
C	836 (67.4%)	744 (60.0%)	*χ* ^2^ = 14.761; *P* ≤ 0.001	1
T	404 (32.6%)	496 (40.0%)		1.38 (1.17-1.63)^∗^
Genotype at rs703752				**1.66 (1.20-2.29)** ^∗^
A/A	7 (1.1%)	2 (0.3%)	*P* = 0.009 (Fisher's exact test)	1
C/A	86 (13.9%)	57 (9.2%)		2.32 (0.47-11.57)
C/C	527 (85.0%)	561 (90.5%)		3.73 (0.77-18.02)
Recessive model at rs703752				
A/A+C/A	93 (15.0%)	59 (9.5%)	*χ* ^2^ = 8.668; *P* = 0.003	1
C/C	527 (85.0%)	561 (90.5%)		1.68 (1.19-2.38)^∗^
Dominant model at rs703752				
A/A	7 (1.1%)	2 (0.3%)	*P* = 0.178 (Fisher's exact test)	1
C/A+C/C	613 (98.9%)	618 (99.7%)		3.53 (0.73-17.05)
Allele at rs703752				
A	100 (8.1%)	61 (4.9%)	*χ* ^2^ = 10.103; *P* =0.001	1
C	1140 (91.9%)	1179 (95.1%)		1.70 (1.22-2.36)^∗^

OR: odds ratio; CI: confidence interval. ^∗^Statistically significant (*a* = 0.05).

**Table 5:** Multivariate analysis of *NKX2.5* gene polymorphism and CHD.

*NKX2.5* gene	B	S.E	Wals	*P* value	Adjusted OR (95% CI)^∗^
Genotype at rs6882776 (control genotype = A/A)					
G/A	0.082	0.169	0.238	0.626	1.09 (0.78-1.51)
G/G	0.018	0.239	0.006	0.940	1.02 (0.64-1.63)
Additive model	0.026	0.114	0.050	0.822	1.03 (0.82-1.28)
Recessive model	0.068	0.161	0.179	0.673	1.07 (0.78-1.47)
Dominant model	-0.031	0.217	0.020	0.886	0.97 (0.63-1.48)
Genotype at rs118026695 (control genotype = T/T)					
C/T	0.766	0.196	15.297	0.001	2.15 (1.47-3.16)
C/C	1.603	0.707	5.147	0.023	4.97 (1.24-19.86)
Additive model	0.774	0.175	19.521	0.001	2.17 (1.54-3.06)
Recessive model	0.821	0.191	18.546	0.001	2.27 (1.56-3.30)
Dominant model	1.450	0.704	4.241	0.039	4.26 (1.07-16.95)
Genotype at rs2277923 (control genotype = C/C)					
T/C	0.477	0.167	8.127	0.004	1.61 (1.16-2.24)
T/T	0.555	0.240	5.352	0.021	1.74 (1.09-2.79)
Additive model	0.328	0.113	8.478	0.004	1.39 (1.11-1.73)
Recessive model	0.496	0.158	9.840	0.002	1.64 (1.20-2.24)
Dominant model	0.302	0.222	1.842	0.175	1.35 (0.88-2.09)
Genotype at rs703752 (control genotype = A/A)					
C/A	0.467	0.970	0.232	0.630	1.60 (0.24-10.68)
C/C	0.893	0.947	0.889	0.346	2.44 (0.38-15.63)
Additive model	0.430	0.212	4.098	0.043	1.54 (1.01-2.33)
Recessive model	0.452	0.229	3.908	0.048	1.57 (1.00-2.46)
Dominant model	0.847	0.946	0.802	0.371	2.33 (0.37-14.90)

OR: odds ratio; CI: confidence interval; SNP: single-nucleotide polymorphism; CHD: congenital heart disease. ^∗^Adjusted for factors with statistical differences in Table 1.

**Table 6:** Interaction analysis of Maternal DM and *NKX2.5* on CHD in offspring.

Maternal DM	Genotype	*β*	*P* value	Adjusted OR (95% CI)^∗^
GDM during this pregnancy	rs118026695			
No	T/T			1
No	C/T	0.804(*β*_g1_)	0.001	2.23 (1.50-3.33)
No	C/C	21.324(*β*_g2_)	0.998	1.823E9
Yes	T/T	1.987(*β*_e1)_	0.001	7.29 (3.29-16.17)
Yes	C/T	2.094(*β*_e1_∗_g1_)	0.008	8.12 (1.71-38.51)
Yes	C/C	0.430(*β*_e1_∗_g2_)	0.620	1.54 (0.28-8.40)
GDM in previous pregnancy experiences	rs118026695			
No	T/T			1
No	C/T	0.861(*β*_g1_)	0.001	2.37 (1.59-3.52)
No	C/C	1.496(*β*_g2_)	0.046	4.46 (1.03-19.44)
Yes	T/T	1.603(*β*_e1)_	0.001	4.97 (2.44-10.11)
Yes	C/T	1.681(*β*_e1_∗_g1_)	0.074	5.37 (0.85-33.87)
Yes	C/C	21.345(*β*_e1_∗_g2_)	0.999	1.862E9
PGDM in the 3 months before this pregnancy	rs118026695			
No	T/T			1
No	C/T	0.826(*β*_g1_)	0.001	2.29 (1.54-3.40)
No	C/C	1.753(*β*_g2_)	0.013	5.77 (1.45-23.05)
Yes	T/T	1.971(*β*_e1)_	0.001	7.18 (3.27-15.74)
Yes	C/T	20.740(*β*_e1_∗_g1_)	0.998	1.017E9
Yes	C/C			
GDM during this pregnancy	rs2277923			
No	C/C			1
No	T/C	0.472(*β*_g1_)	0.007	1.60 (1.14-2.26)
No	T/T	0.703(*β*_g2_)	0.006	2.02 (1.22-3.34)
Yes	C/C	1.984(*β*_e1)_	0.001	7.27 (2.37-22.33)
Yes	T/C	2.875(*β*e1∗g1)	0.001	17.72 (5.79-54.24)
Yes	T/T	0.338(*β*_e1_∗_g2_)	0.628	1.40 (0.36-5.50)
GDM in previous pregnancy experiences	rs2277923			
No	C/C			1
No	T/C	0.628(*β*_g1_)	0.001	1.88 (1.33-2.65)
No	T/T	0.715(*β*_g2_)	0.004	2.05 (1.25-3.34)
Yes	C/C	3.255(*β*_e1)_	0.001	25.92 (7.20-93.26)
Yes	T/C	1.225(*β*_e1_∗_g1_)	0.005	3.41 (1.44-80.07)
Yes	T/T	0.867(*β*_e1_∗_g2_)	0.317	2.38 (0.44-12.99)
PGDM in the 3 months before this pregnancy	rs2277923			
No	C/C			1
No	T/C	0.579(*β*_g1_)	0.001	1.79 (1.27-2.52)
No	T/T	0.644(*β*_g2_)	0.009	1.90 (1.17-3.09)
Yes	C/C	3.592(*β*_e1)_	0.001	36.30 (7.62-172.98)
Yes	T/C	1.421(*β*_e1_∗_g1_)	0.002	4.14 (1.67-10.27)
Yes	T/T	21.312(*β*_e1_∗_g2_)	0.999	1.802E9

DM: diabetes mellitus; CHD: congenital heart disease; GDM: gestational diabetes mellitus; PGDM: pregestational diabetes mellitus; OR: odds ratio; CI: confidence interval. ^∗^Adjusted for factors with statistical differences in Table 1.

## Data Availability

The data used to support the findings of this study are included within the article.
